# Convergence in parameters and predictions using computational experimental design

**DOI:** 10.1098/rsfs.2013.0008

**Published:** 2013-08-06

**Authors:** David R. Hagen, Jacob K. White, Bruce Tidor

**Affiliations:** 1 Department of Biological Engineering, Massachusetts Institute of Technology, Cambridge, MA 02139, USA; 2 Computer Science and Artificial Intelligence Laboratory, Massachusetts Institute of Technology, Cambridge, MA 02139, USA; 3 Department of Electrical Engineering and Computer Science, Massachusetts Institute of Technology, Cambridge, MA 02139, USA

**Keywords:** systems biology, ordinary differential equation modelling, parameter uncertainty, Fisher information, optimal experimental design

## Abstract

Typically, biological models fitted to experimental data suffer from significant parameter uncertainty, which can lead to inaccurate or uncertain predictions. One school of thought holds that accurate estimation of the true parameters of a biological system is inherently problematic. Recent work, however, suggests that optimal experimental design techniques can select sets of experiments whose members probe complementary aspects of a biochemical network that together can account for its full behaviour. Here, we implemented an experimental design approach for selecting sets of experiments that constrain parameter uncertainty. We demonstrated with a model of the epidermal growth factor–nerve growth factor pathway that, after synthetically performing a handful of optimal experiments, the uncertainty in all 48 parameters converged below 10 per cent. Furthermore, the fitted parameters converged to their true values with a small error consistent with the residual uncertainty. When untested experimental conditions were simulated with the fitted models, the predicted species concentrations converged to their true values with errors that were consistent with the residual uncertainty. This paper suggests that accurate parameter estimation is achievable with complementary experiments specifically designed for the task, and that the resulting parametrized models are capable of accurate predictions.

## 1. Introduction

A goal of systems biology is to construct models that incorporate known mechanisms and reflect existing data under laboratory conditions. The notion is that mechanistic mathematical models not only recapitulate existing measurements but can also ultimately predict the behaviour of modelled systems under novel conditions not previously tested and be the basis of design work as is done in more mature fields of engineering [1–6]. In addition, high-quality, mechanistically accurate models can also lead to novel insights into systems operations. Biological systems are sufficiently complex that mechanistic models will contain large numbers of parameters and thus will require correspondingly large quantities of data for training. Recent and future advances in the development of high-throughput measurement techniques (e.g. mass spectrometry [7] and flow cytometry [8]) continue to increase the quantity and quality of data collected, and bring nearer the promise of meeting the needs of true mechanistic understanding of biological complexity, as reflected in the ability to determine the topology and parameters of corresponding models. Important research areas include the development of experimental design strategies to efficiently deploy experiments to probe new aspects of their operation, computational framing of the space of relevant models and probabilistic treatments of model uncertainty. Here, we focus on the first of these areas.

Recent work by Gutenkunst *et al.* [9] has suggested that it is difficult, if not impossible, to accurately estimate the parameters of a typical biological model, regardless of how accurately the data are collected, how many species of the model are simultaneously measured or how finely the species are measured in time. It was found that, for typical biological models under typical experimental conditions, there were some directions in parameter space that had so little effect on the measured quantities that the resulting uncertainty in many of the parameters was too vast to be overcome by higher quality measurements. In later work [10–12], however, our group showed that the seemingly vast parameter uncertainty could be dramatically reduced with a relatively small number of carefully selected perturbation experiments. We demonstrated that sets of experiments could be found that together exercised the epidermal growth factor (EGF) and nerve growth factor (NGF) pathways in sufficiently complementary ways so as to allow all parameters to be determined within 10 per cent uncertainty. This proof-of-concept study highlighted a potential role for computational design of experimental conditions to efficiently reduce parameter uncertainty.

**Figure 1.**
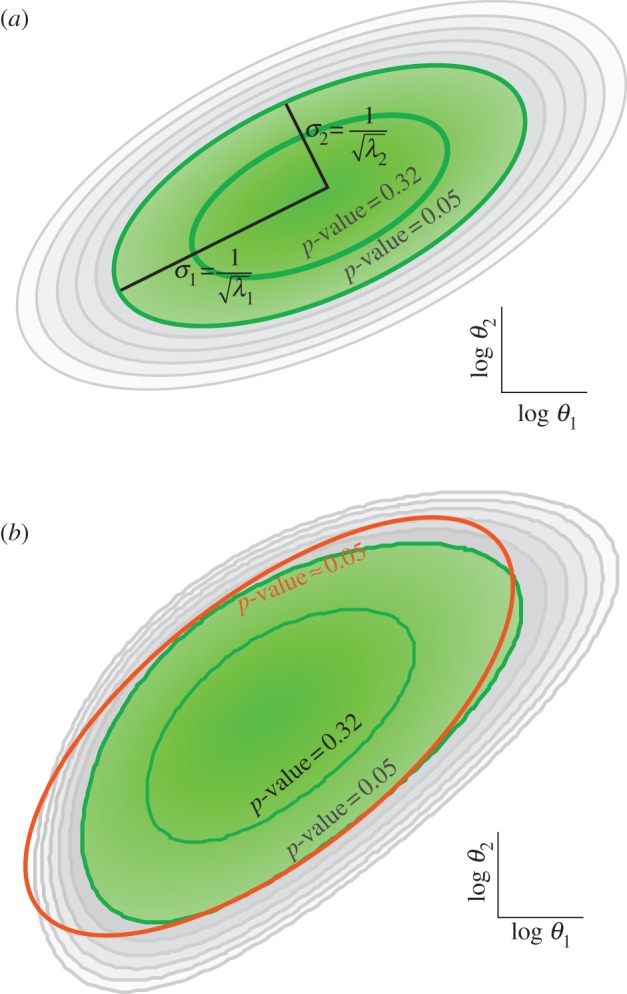
Nonlinearity. (*a*) In a linear model, the Fisher information matrix exactly describes the likelihood of the parameter sets in the neighbourhood of the most likely parameters. This likelihood is Gaussian, which has contours that are ellipsoids in parameter space. (*b*) The likelihood of the parameters of biological models is not exactly Gaussian. For two parameters of the EGF–NGF model fitted to a nominal experiment, it can be seen that the true contours for the likelihood of the association and dissociation parameters (green line) are only approximated by the linearization (orange line). All contours in both plots represent parameter sets of equal likelihood.

Our previous work effectively demonstrated the existence in principle of a sequence of experiments that progressively reduce parameter uncertainty to manageable levels; it did not, however, investigate whether the sequence of experiments might be discoverable in a practical setting. In an effort to address the challenge of parameter error reduction issued by Gutenkunst *et al.* [9], most aspects of our study paralleled theirs, and these choices precluded drawing conclusions regarding the practicality of parameter error reduction through our scheme. These limitations included (i) the actual model parameters were known and used at each stage of the experimental design progression to select the next experiment in the sequence, but, in any real application, the actual model parameters would be unknown; (ii) the data measurements in each experiment provided the average information that could be obtained from any species at any time, but, in practical situations, each data point provides information from a single species at a discrete time; and (iii) the model was assumed to be linear in the sense that the Fisher information matrix was assumed to accurately represent the parameter uncertainty, whereas, in practice, the Fisher information matrix is just the first (linear) term in an expansion of that error (figure 1). The current report addresses the practicality of setting up and solving as an optimization problem the task of selecting experiments to progressively reduce parameter uncertainty in biological models by removing these limitations and seeking convergence to the true, unknown parameters. In particular, the performance of the approach could degrade significantly because best-fit parameters with their inherent errors, rather than perfect parameters, are used in the experimental design phase. A major result of the work presented here is that fitted parameters do, indeed, perform well in this role.

For comparison with previous work from our group and that of Sethna, we carried out this study with the same model of the EGF and NGF receptor kinase cascades, which are an important pair of interlaced signalling networks in mammalian cells [13]. The EGF receptor pathway, in particular, has become one of the best-studied signalling pathways in biology [14–17]. Constitutive activation of this pathway is associated with cancers of the breast, bladder, cervix, kidney, ovary, lung and other tissues. Despite nearly half a century of investigation, much remains unknown about this pathway [18,19]. A number of models have been developed, differing in the species included, the connections among them and the data with which they were parametrized. The diversity of the available models of this system reflects the underlying uncertainty concerning the true nature of these pathways, in terms of both topology and parameters [13,20–32]. The EGF–NGF model used in this work is an ordinary differential equation (ODE) model developed by Brown *et al.* [13] in which the enzymatic reactions of the network are modelled with Michaelis–Menten kinetics.

We used synthetic datasets generated according to the published model and showed that the uncertainty in all 48 parameters can be effectively reduced below 10 per cent using the discrete data generated from a small set of complementary experiments chosen according to a greedy optimization algorithm. The parameters estimated by fitting to the data of these chosen experiments converged to their true values with a residual error consistent with 10 per cent uncertainty. Furthermore, the error in the predictions made according to these parameters was consistent with 10 per cent parameter uncertainty.

## 2. Methods

### 2.1. Scenario overview

In our scenario, we treated the published model as the true system. To perform a synthetic experiment, this model was simulated according to the defined experimental conditions, and noisy data were generated according to the measurement scheme of that experiment by adding Gaussian random noise corresponding to 10 per cent measurement error. As explained in more detail below, simulated data were fitted to the topology of the model but without access to the true parameters, which resulted in trial parameters and uncertainty represented by the Fisher information matrix. Experiments were designed to reduce uncertainty, and the process was continued with these experiments implemented in the model.

A nominal experiment was performed, and a starting model was fitted to the resulting data. A nominal Fisher information matrix was computed. Using the fitted model, the expected information matrices for a large set of candidate experiments were computed. The nominal information matrix was added to each of the expected information matrices to predict the combined information matrix after doing each of the candidate experiments. The utility of each sum was quantified using a goal function, and the highest-ranked experiment was selected.

The selected experiment was performed, using the true model to generate noisy measurements in accordance with the experiment's design. The model was fitted to the union of the nominal dataset and the new dataset from the best experiment. This fitting returned a new parameter set from which the expected information matrices were recomputed, and the subsequent best experiment was selected. This procedure of computing, selecting, performing and fitting was repeated iteratively until all the parameter directions had uncertainties below 10 per cent.

### 2.2. The model

The model describes signalling from the EGF and NGF receptors in rat PC12 cells and was developed by Brown *et al*. [13]. It has 32 species and 48 parameters for 24 reactions in two compartments. We obtained a version encoded in the systems biology mark-up language from the BioModels database [33]. The model includes two extracellular species, EGF and NGF, which each bind to the corresponding receptor to form two complexes. The remaining species are divided between 11 enzymes that can exist as either an active or inactive species and four enzymes that are constitutively active. The parameters are divided into three classes: (i) four rate constants for ligand–receptor association and dissociation, (ii) 22 *k*_cat_ values, and (iii) 22 *K*_m_ values of the Michaelis–Menten enzymatic reactions. The species, reactions and rate parameters were retained from the original model. An illustration of the model topology is provided in the electronic supplementary material, figure S1, and a list of parameters and their values is available in the electronic supplementary material, table S1. The extracellular compartment was given a volume of 1000 times that of the intracellular compartment to reflect the modification made by Apgar *et al*. [10]. The starting model had the topology of the true model, but each parameter was set to the geometrical mean of the class of parameters to which it belonged.

### 2.3. The experiments

We defined a battery of candidate experiments that served as a collection of different conditions and perturbations to the system, a selection of which could potentially drive a sufficiently wide variety of system behaviour to allow definition of most or all of the parameters with small uncertainty. Each experiment included stimulation with one of five discrete values of EGF (1 × 10^7^, 1 × 10^5^, 1 × 10^3^, 1 × 10^1^ and 0 molecules per cell) and five discrete values of NGF (4.56 × 10^7^, 4.56 × 10^5^, 4.56 × 10^3^, 4.56 × 10^7^ and 0 molecules per cell). In this model, 1 ng ml^−1^ is equal to 1000 molecules per cell of EGF and 4560 molecules per cell of NGF. In addition, up to three proteins, in the network, could have their concentrations changed through knock-down or over-expression. The species that could be changed were the two receptors, the 11 inactive enzymes and the four constitutively active enzymes, all of which started with non-zero concentrations as their initial condition. To represent knock-down or over-expression, each of these species had its initial concentration decreased or increased by a 100-fold of its nominal value, respectively. Considering all combinations of EGF and NGF concentrations and knock-downs and over-expressions, there were 150 475 possible experiments. The nominal experiment was the one with an initial EGF concentration equal to 1000 molecules per cell (1 ng ml^−1^) and an initial NGF concentration equal to 4560 molecules per cell (1 ng ml^−1^) and no knock-downs or over-expressions.

All experiments were performed synthetically by simulation of the system for 120 min using the numerical ODE solver ode15s, with analytical Jacobian supplied, in Matlab (2008b, The MathWorks, Natick, MA, USA). Each experiment called for measuring all 32 species at 100 evenly spaced time points, and all data points were subjected to random Gaussian noise with a standard deviation of 10 per cent or one molecule, whichever was larger. The experimental conditions and measurement scheme are comparable to those used by Apgar *et al*. [10].

### 2.4. Data fitting

The fit of the model to data for any particular parametrization was quantified using generalized least squares,2.1
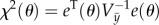
and2.2
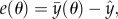
where 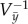 is the variance–covariance matrix of the measurements, **θ** is the vector of parameters of length *n_*θ*_* and *e*(**θ**) is the difference between the model predictions 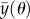 and the data points 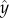. 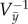 is a square symmetric positive semi-definite matrix with a size equal to the number of measurements *n*, and *e*(**θ**), *y*(**θ**) and 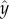 are all vectors of length *n*. Our procedure assumed that 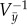 was a constant for the dataset. For the example in this paper, there was no covariance proper, so 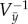 was diagonal, and we estimated the uncertainty as 10 per cent of the value of each data point or one molecule, whichever was larger.

The best-fit parameters were defined as the vector **θ** that minimized **χ**^2^. This nonlinear optimization was accomplished using the active-set algorithm implemented within fmincon in Matlab. The lower bound of the search space was 1000-fold less than the smallest value in each class of parameters in the published model; the upper bound was 1000-fold greater than the largest value in each class.

### 2.5. Information

The Fisher information matrix was used to quantify the knowledge of the parameters. The Fisher information matrix *F*(**θ**) for a set of normalized parameters that affect the means of a multivariate normal distribution is given by2.3
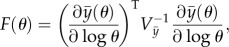
where 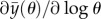 is the sensitivity of the values of the data points to the normalized parameters (an *n* × *n_*θ*_* matrix calculated by integrating the forward sensitivities together with the system during simulation using the numerical ODE solver ode15s in Matlab).

To compute the information matrix for candidate experiments, the model was simulated for each candidate experiment using the fitted parameters. The uncertainty of each measurement was computed according to the measurement scheme of the candidate experiment. The sensitivity of each concentration to the parameters was also integrated. Using equation (2.3), the measurement uncertainty and sensitivity were used to compute the information matrix from the expected results of a candidate experiment. When the information matrix was computed in this way, it was called an *expected* information matrix.

### 2.6. Parameter goal

Three different goal functions were used to evaluate the efficiency of candidate experiments. Each of the goal functions was based on the eigenvalues resulting from an eigendecomposition of the information matrix. The inverse square roots of these eigenvalues correspond to the uncertainties in the eigendirections of parameter space [34,35]. The first goal function maximized the number of eigendirections whose uncertainties were less than 10 per cent and used the remaining directions to break ties,2.4
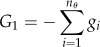
and2.5
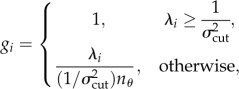
where **λ*_i_* is the *i*th eigenvalue of the information matrix and **σ**_cut_ is 0.1, the uncertainty cut-off value. This goal function is equivalent to that used by Apgar *et al.* [10].

The second goal function minimized the sum of the natural logarithm of the ellipsoid axes. It is equivalent to minimizing the volume of the uncertainty ellipsoid, as well as minimizing the entropy of the parameter probability distribution, as well as maximizing the determinant of the Fisher information matrix,2.6
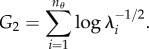


The third goal function was identical to the second except that no additional advantage was given to directions that were tighter than 10 per cent. In other words, directions that had errors lower than 10 per cent contributed to the goal function the same as if they were exactly 10 per cent,2.7
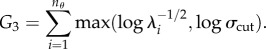


## 3. Results and discussion

**Figure 2.**
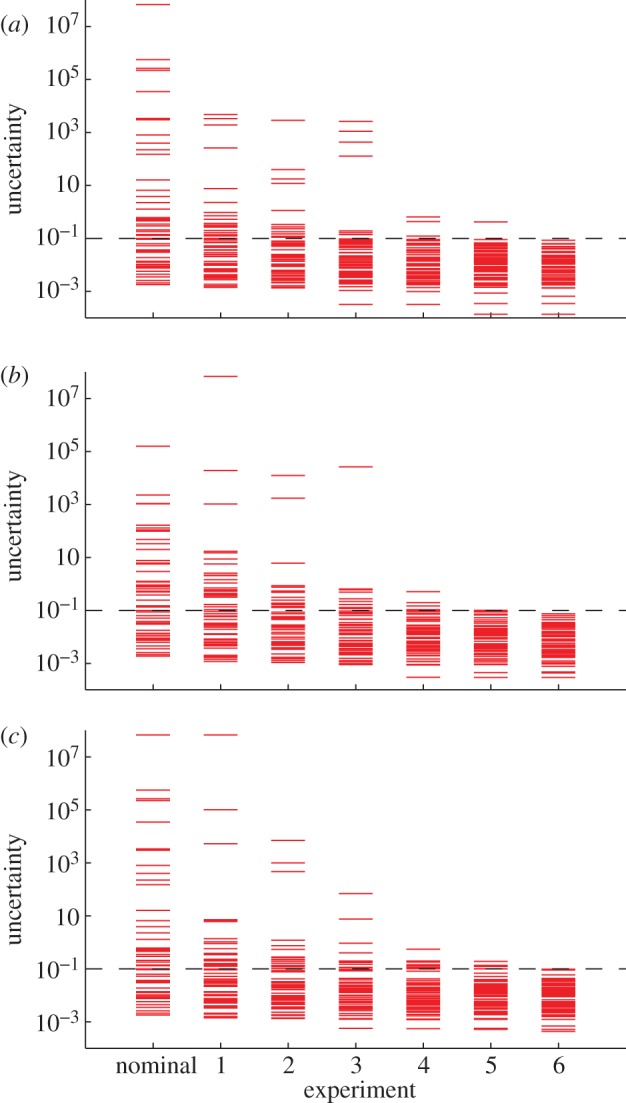
Parameter uncertainty. Progressively choosing the optimal experiment eventually led to all 48 parameter direction uncertainties being less than 10%. In all three cases, it took six additional experiments. It does not appear that the rate of convergence was strongly influenced by the particular goal function used.

**Figure 3.**
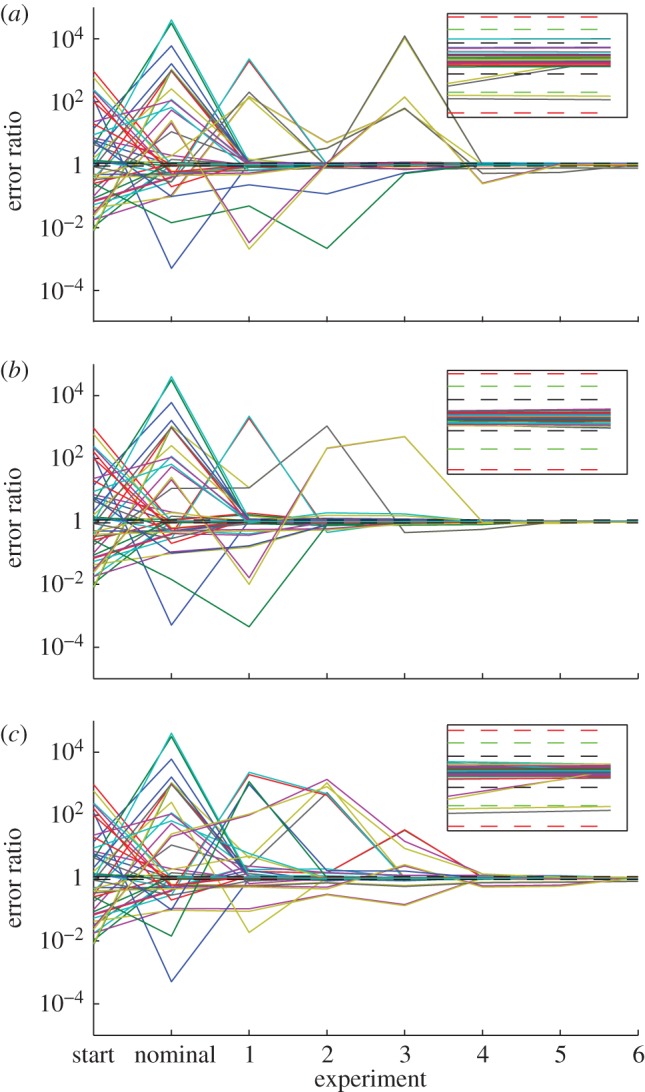
Parameter error. Here is shown the ratio of the fitted parameters to the true parameters after each iteration of optimally chosen experiments according to the three goal functions. ‘Start’ is the scrambled parameter set before the nominal experiment is fitted. Each inset shows a magnified view of the final parameter set error. The black, green and red dashed lines are 10%, 20% and 30% error, respectively. By the final experiment, all parameter errors are less than 30% and most parameter errors are less than 10%. This suggests that the linear approximation of the uncertainties is an appropriate estimate of the underlying parameter error when the data are plentiful and the uncertainties are small.

We began our parameter estimation experiments with no knowledge of the actual model parameters. Instead, the process was begun with simulated experimental data with 10 per cent added noise from a nominal experiment (intact network stimulated with 1000 molecules (1 ng ml^−1^) of EGF and 4560 molecules (1 ng ml^−1^) of NGF per cell). Initial parameters were estimated by fitting to the data according to weighted least squares, and the corresponding Fisher information matrix was computed. The spectra of inverse-square-root eigenvalues 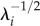, also referred to as the uncertainties in parameter eigendirections, is given on the far left column of figure 2 (marked nominal). The three panels of figure 2 correspond to the three goal functions used. The nominal spectra show some parameter directions to be well determined (uncertainties below 10%) but others to be poorly determined (uncertainties greater than 1000-fold). The actual errors in each parameter are shown on the left in figure 3 (marked nominal) and are consistent with the uncertainties.

A large collection of 150 475 candidate experiments that included a variety of levels of stimulation with EGF and NGF acting on versions of the network modified with up to three expression changes (100-fold over-expression or under-expression each) was evaluated to determine which would lead to the largest reduction in parameter uncertainty. The selected experiment was simulated and 10 per cent noise was added to the resulting concentrations to produce simulated experimental data. New parameters were fitted using cumulative data from the simulated experiments, the corresponding updated Fisher information matrices were computed, and the process was repeated iteratively. The resulting uncertainties and parameter errors are given in figures 2 and 3. The selection and implementation of experiments proceeded until all parameter uncertainties were below 10 per cent. The entire procedure was carried out three times in parallel for the three different goal functions used, with similar results.

The sequential addition of experiments selected in the greedy optimization progressively reduced parameter uncertainty. Uncertainty was very low after just three experiments beyond the nominal experiment, and all parameters were determined to within 10 per cent with six experiments beyond the nominal.

An important aspect of the work is that the approximate parameters fitted after each new experiment were used to compute the Fisher information matrices leading to the selection of the following experiment. Figure 3 shows that, early in the procedure, many of the estimated parameters were significantly in error, yet they still led to the selection of experiments that efficiently reduced parameter uncertainty. This is significant in light of the fact that the theory strictly applies only to linear models. Even though there are substantial nonlinearities in the EGF–NGF model, the behaviour of the parameter uncertainties is similar to that expected from the linear theory. After the final experiment, nearly all 48 parameters were correct to within 10 per cent error. On average, two parameters were outside 10 per cent, roughly one was outside 20 per cent and none were outside 30 per cent. This is consistent with the linearized uncertainties from which it is expected that no more than 33 per cent and 5 per cent of parameters should be outside 1 and 2 s.d., respectively (keeping in mind that many of the parameters had less than 10% uncertainty by the end of the procedure). Taken together, the results show that the estimated parameters converged to the true parameters, and the residual parameter errors were consistent with the calculated uncertainties. This correspondence lends confidence to the usefulness of the method for cases where the actual parameters will be unknown.

The experiments selected are shown in table 1. Three genetic perturbations were used for each experiment except for the final experiment for goal functions 1 and 3. When comparing the species knocked-down and over-expressed with the parameters subsequently determined, there appears to be a relationship where determining the parameters of reaction is helped by changing the concentration of the enzyme or especially the substrate of that reaction (see the electronic supplementary material, figures S2–S4). Because the average information provided by one experiment alone is the same for zero, one, two or three perturbations (see the electronic supplementary material, figure S5), the apparent preference for three perturbations is probably due not to an inherently greater sensitivity to the parameters but instead to the greater diversity (and thus, perhaps complementarity) that three perturbations allow as well as the fact that three-perturbation experiments make up 90 per cent of the candidate experiment pool. One anonymous reviewer noted that, for the first goal function, there was never chosen an EGF-dominated stimulation, suggesting that reasonable parameter estimation may be achievable without broad exploration of the input concentration space. It remains to be seen how much of the input space could be removed while still retaining the ability to determine the parameters well within a small number of experiments. Table 1 also shows that different sets of experiments were used in the three parallel runs using different goal functions. Based on our previous work, we hypothesize that what is important about the sets of experiments is their internal complementarity [10]. Multiple sets of experiments can efficiently inform about all 48 parameters, but a set must still be self-complementary to be informative about all parameters. For example, sets of six experiments constructed by mixing two experiments from each of the three complementary sets in table 1 were significantly less informative about the parameters than the optimized sets in table 1 (see the electronic supplementary material, figure S6).

**Table 1.** Optimal experiments. The experiments chosen according to each of the three goal functions are shown here. The scenarios began with fitting to a nominal experiment. In each case, it required six additional experiments to constrain the parameter uncertainty below 10%. All optimal experiments knocked-down or over-expressed the maximum of three proteins in the network, with the exception of the final experiments in the cases of goal functions 1 and 3.

experiments	EGF (molecules per cell)	NGF (molecules per cell)	knocked-down	over-expressed
goal function 1; count of uncertainties above 10%				
nom.	1000	4560		
1	0	4560	RasGap, Erk	Rap1
2	10	4.56 × 10^7^	RapGap	Erk, Akt
3	0	4560	Raf1PPtase	Mek, C3G
4	1.00 × 10^5^	4.56 × 10^7^		Sos, Raf1, Braf
5	0	4.56 × 10^7^		Braf, C3G, Rap1
6	1.00 × 10^7^	4.56 × 10^7^		Sos, Ras
goal function 2; sum of log of uncertainties				
nom.	1000	4560		
1	1.00 × 10^7^	4560	EGFR, P90Rsk	Rap1
2	10	4.56 × 10^5^	RasGap, Raf1PPtase	Erk
3	1.00 × 10^5^	45.6		Sos, Mek, PI3K
4	0	4560	RapGap	Braf, Rap1
5	1.00 × 10^7^	4560	RasGap	Ras, Raf1
6	1000	4.56 × 10^7^	Rap1	PI3K, Akt
goal function 3; sum of log of uncertainties floored at 10%				
nom.	1000	4560		
1	0	4.56 × 10^7^	RasGap	Raf1, Rap1
2	1.00 × 10^5^	45.6	Erk, RapGap	Braf
3	10	4.56 × 10^5^	RasGap	Ras, Mek
4	1.00 × 10^7^	4.56 × 10^7^	Raf1PPtase	Sos, Braf
5	10	4560		Braf, C3G, Rap1
6	10	4560		Erk, Akt

In a linear system, the outputs change linearly in response to a change in the parameters. Therefore, when a system has non-negative outputs for any non-negative parameters, the per cent error in any output is bounded by the worst per cent error in the parameters. With a nonlinear system, this guarantee disappears. To examine the effect that the optimal experiments had on the prediction error of the EGF–NGF model, we compared the predictions of the three final fitted models with the predictions of the true model. We simulated the model under all 150 475 candidate experimental conditions using both the true parameters and the final estimated parameters and sampled the species according to the standard scheme. No noise was added to the measurements, so that we quantified only the component of the error that came from incorrect parametrization. We computed the relative error in each prediction,3.1
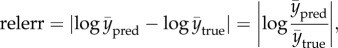
where 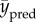 is the predicted species concentration, and 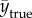 is the actual species concentration according to the true model. Predicted or actual concentrations less than one molecule per cell were not considered.

**Figure 4.**
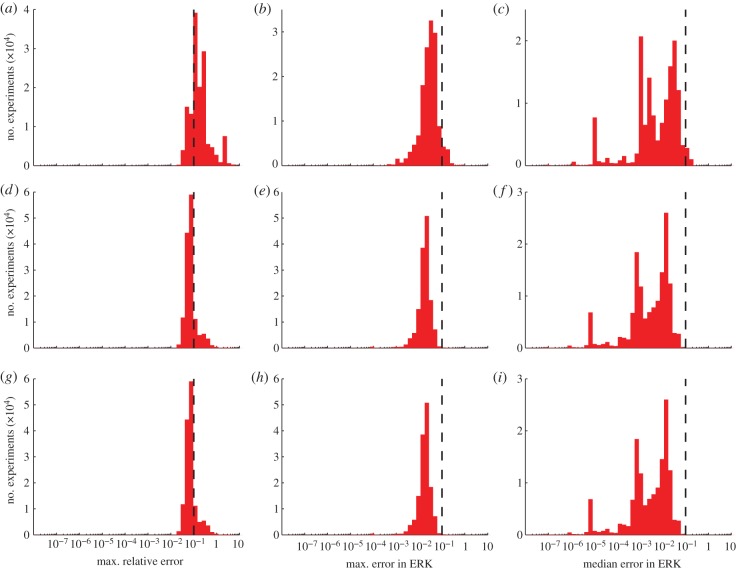
Final prediction errors. For all 150 475 experiments, we computed the relative error of all data points, by comparing the predictions of the final fitted model with the true model. The error in each experiment was summarized in three ways: the maximum relative error in any species at any time; the maximum relative error in active ERK at any time; and the median relative error in active ERK over all time. Here, each column of plots is a different summary and each row is a different goal function. (*a–c*) For goal function 1; (*c–e*) for goal function 2; and (*g–i*) for goal function 3. (*a,d*,*g*) The overall maximum relative error results; (*b,e,h*) the maximum ERK relative error results; and (*c,f*,*i*) the median ERK relative error results. As expected, the worst error in all species clusters around 10% (dashed black line), consistent with parameter uncertainty of about 10%. Some errors are much worse (approx. 100%), though this is dominated by transient intermediate species, as the worst error in active ERK, the output of the system, is much smaller and almost exclusively confined below 10%. (Online version in colour.)

Because there were 3200 data points in each experiment (32 species, 100 data points each), we summarized the prediction error in each experiment in three ways: (i) the largest prediction error of all the data points in a given experiment, (ii) the largest of all ERK prediction errors, and (iii) the median of all ERK prediction errors. The first summary is the most conservative, but the second and third may be the most informative, because it is often the prediction error of a specified output, not of all intermediate species, that is of greatest interest. A histogram of the prediction errors according to these three summaries from the 150 475 candidate experiments can be seen in figure 4. The worst prediction errors cluster around 10 per cent, which is consistent with the parameter uncertainties and parameter errors. Taken together, the results show that the parameters converged to their true values as data from optimal experiments were added (figure 3), and the residual prediction error in yet-to-be-done experiments was consistent with the calculated parameter uncertainty (figure 4). While entirely expected for linear models, it is gratifying to see similar results in this nonlinear system representative of the types of signalling models currently used in systems biology.

**Figure 5.**
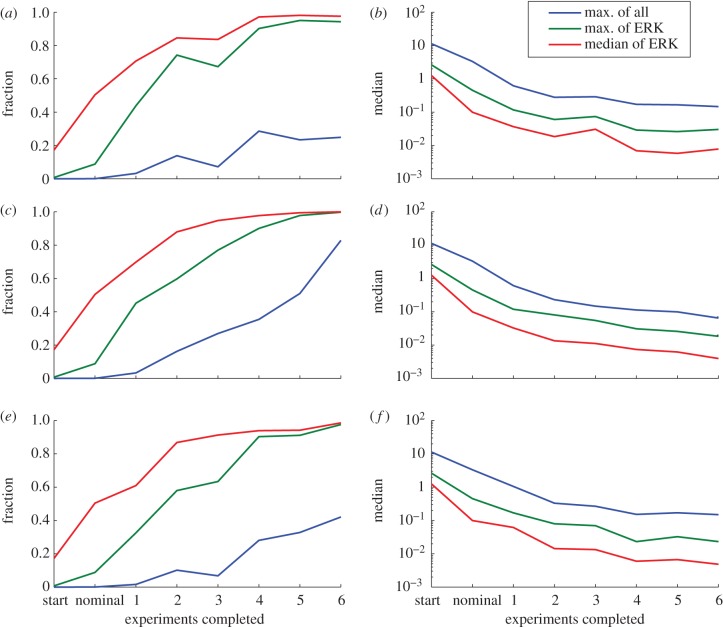
Prediction errors. As the model was fitted to an increasing number of experiments directed by the three goal functions, we summarized the prediction errors in the set of candidate experiments compared with the true model according to the three summaries described in figure 4: the worst error in all species (blue), the worst error in active ERK (green), and the median error in active ERK (red). (*a*,*b*) For goal function 1; (*c*,*d*) for goal function 2; (*e*,*f*) for goal function 3. These plots show the fraction of the experiments whose error was less than 10% (*a,c*,*e*) and the median error over all experiments (*b,d*,*f*). The prediction errors generally improve with each experiment, regardless of the goal function used or the technique used to summarize the error. The early optimally chosen experiments appear to improve the prediction more than final experiments.

In addition to the prediction error after the final iteration, we also examined the prediction error from the fitted models at intermediate iterations. The predictions of many experiments made by the models only fitted to three optimal experiments were worse than 10 per cent (see the electronic supplementary material, figure S7). For each of the three error summary types at each iteration, we further summarized the histograms in two ways: (i) the fraction of experiments whose errors were below 10 per cent and (ii) the median error over all experiments. Figure 5 shows the summarized prediction errors of the models after each experiment was added. As expected, the predictions by all measures improved nearly monotonically with increasing data. The improvements in the predictions tended to taper off with the last few experiments; that is, the experiments chosen to determine the most difficult parameters did not as greatly improve the global predictions. This is consistent with the findings of Apgar *et al.* [10], who showed that the few final parameters determined by the greedy search algorithm had only a few experiments that were sensitive enough to these parameters to determine them. The converse, that there are only a few conditions whose outcome is strongly affected by finally determined parameters, is shown here.

## 4. Conclusion

Our previous work demonstrated that optimal experimental design could select experiments that sequentially reduced parameter uncertainty, but it was a theoretical demonstration that made use of the ideal model parameters, which, in practice, are unknown [10]. Here, we extend those findings by demonstrating convergence to the correct parameters through iterative estimation and experimental design cycles, without knowledge of the actual parameters. The parameter uncertainties converged to below 10 per cent for all parameter directions, and the actual parameter errors of the final models were consistent with this uncertainty level. Moreover, the prediction errors on experiments not used in the parametrization were also small and consistent with the parameter errors. This is an important demonstration because, at each stage, the scenario essentially needs to identify measurements that are sensitive to the poorly known parameters based on a linearization about a set of parameters that could still have large errors. If the model were purely linear, this would not be a concern; for nonlinear models, the parameter error means the linearization could frequently be taken about a point significantly different from the true parameter set, and so the linearization would be grossly inaccurate. A major result of this work is that these inaccuracies do not spoil the rapid convergence of the method. We note that the increase from five experiments in our previous work to the current six is due to the use of discrete data here rather than due to continuous data as used previously; we determined this from the observation that even using perfect parameters with discretely drawn data requires six experiments to provide all parameters with 10 per cent uncertainty (data not shown). Using six randomly chosen experiments or a single experiment with all the perturbations of a set of optimal experiments did not reduce parameter uncertainty as effectively as the set of experiments chosen by our optimization scheme (see the electronic supplementary material, figures S8–S10).

Our method can be applied to any model defined as a system of ODEs, including those that model multi-stable systems and oscillating systems, as well as those with multiple minima when parameter fitting. In models with these properties, the nonlinearity is even more apparent than in the EGF–NGF model. Linearization was an appropriate approximation for our test system, but further study will be required to determine whether this continues to hold for other biological systems with more complex behaviours.

As close as we tried to make our synthetic procedure mirror a real example, there are a number of real system traits that were neglected. Most obviously, a real system is not a set of ODEs. Real biomolecular systems are confined within cells and subject to stochastic effects. Because the number of molecules in our test system was mostly in the hundreds of thousands, stochasticity would be expected to be only a small perturbation. But, many biological systems operate with only a few molecules of some species per cell, notably if DNA is involved, which usually exists as one or two copies per cell. Stochasticity could mitigate our conclusions in three ways: (i) even a perfectly parameterized ODE may not accurately recreate the behaviour of a stochastic system, (ii) aggregate measurements over many cells may not reflect the behaviour of any single cell and, thus, fitting to such data could be meaningless or inappropriate, and (iii) predicting the next best experiment requires integrating the sensitivities of the species to the parameters, and it is unclear how meaningful these calculations would be in systems where stochasticity was dominant. One way to deal with systems in which stochasticity is important is to use a deterministic approximation to the stochastic noise, such as the linear noise approximation [36] or mass fluctuation kinetics [37]. The Fisher information matrix has been derived for the linear noise approximation and has been used for optimal experimental design [38].

We also did not consider any uncertainty in the inputs to the model. For example, the knock-downs were assumed to flawlessly reduce the initial concentrations to one-hundredth of their prior value. This cannot be done in reality, but a straightforward extension of the method would propagate the uncertainty in the inputs to the uncertainty in the outputs. In lieu of propagating input uncertainty, we repeated the procedure for the first goal function where, instead of the knock-downs and over-expressions being perfectly effective at making 100-fold changes, the effect of the knock-down and over-expression in the true experiments was determined randomly between one- and 1000-fold on a logarithmic scale. Despite the fact that some knock-downs and over-expressions could have nearly no effect and that the optimal experimental design algorithm had no knowledge of this possibility when selecting the next best experiment to do, this only increased the number of experiments needed to 10 (see the electronic supplementary material, figures S11 and S12). The experiments chosen and the actual perturbations used are available in the electronic supplementary material, table S2.

Alternative methods for minimizing the uncertainty in biological models through optimal experimental design have been previously described. Some methods adopt a rigorous Bayesian approach [39,40]. Other methods adopt a different control mechanism, such as time-point selection [41] and dynamic input control [42,43]. The method investigated here, which selects experiments from a discrete set, is complementary to these alternative control mechanisms. One could imagine combining methods to first select the optimal general conditions from a discrete set and then using time-point selection to remove non-informative measurements and altering the dynamic input profile to further enhance the information gained from a possible experiment. Existing methods also vary in terms of their goal, such as the various optimality functions of the covariance matrix [44] and minimizing prediction uncertainty [45]. All three of our goal functions operated on the parameter covariance matrix and were designed to minimize uncertainty in parameter eigendirections. In fact, goal function 2 is equivalent to the popular D-optimal criterion of maximizing the determinant of the Fisher information matrix. Seeking to maximize the trace of the Fisher information matrix (T-optimality) or minimize the trace of the covariance matrix (A-optimality) are popular goals that we did not test. Operating on prediction uncertainty instead may be preferable if knowledge of the system is a secondary goal to using the model for a specialized task.

It should be noted that having a diverse set of candidate experiments is critical to the successful outcome of this procedure. This method selects the best experiment but does not design new experiments should the candidate set be insufficient to find all the parameters. As indicated by the computation of the Fisher information matrix, good experiments are those that make measurements that are sensitive to the yet unknown parameters. If there are portions of the network that cannot be differentially perturbed with existing techniques, it may not be possible to discover the values of the parameters important there. If the number of time points per experiment is reduced from 100 to 80, 40, 20, 10 or 5, the parameters can still be determined, though it takes a few more experiments to do so (up to 12 experiments; electronic supplementary material, figure S13). Furthermore, if the measurement uncertainty is increased from 10 per cent to 20 per cent, it still takes only six additional experiments to determine all parameters better than 10 per cent, reinforcing the importance of complementarity. But when the measurement uncertainty is increased to twofold, it now takes 22 experiments, although the number of experiments needed can be brought back down by reducing the desired parameter uncertainty to twofold as well (see the electronic supplementary material, table S3).

Finally, our method assumes that the topology is already known. This can be far from true. Even with an incorrect topology fitted to data, it is possible to use our approach and predict the best experiment to minimize parameter uncertainty. Yet it is unclear what the ultimate result of that would be. Would the experiments actually be effective at minimizing parameter uncertainty? Would it choose experiments that eliminated it as a possible topology? Would the experiments it chose still be best, or nearly best, once the correct topology was found?

Akt is known to also downregulate B-Raf, a reaction that is not described in this model. We added a reaction to the original model in which Akt downregulated B-Raf with Akt's normal *K*_m_ and a *k*_cat_ of one-hundredth of the strength by which Akt influences Raf-1. We used this modified model as the true model to generate data, while using the published model to actually fit to the data. This was intended to represent one case where the model topology does not match the real system. The model was able to fit the data of the nominal experiment, and two optimally chosen experiments according to goal function 1. However, the model failed to fit the data after the third experiment according to a *χ*^2^ test between the data and the best-fit model (data not shown). This suggests that computational experimental design can not only lead to well-determined parameters for appropriate topologies but can also lead to indications that topologies are insufficient to explain observed data.

We are currently working on methods to discover topology-defining experiments that can be used together with the method presented here to discover parameter-defining experiments so that models can be improved progressively along both directions.
